# Thromboelastography (TEG) in normal pregnancy and its diagnostic efficacy in patients with gestational hypertension, gestational diabetes mellitus, or preeclampsia

**DOI:** 10.1002/jcla.23623

**Published:** 2020-10-17

**Authors:** Xin Xie, Meng Wang, Yifan Lu, Jiazi Zeng, Jing Wang, Chunhong Zhang, Hongyuan Zhu, Yujie Song, Lican Han, Ying Liu, Jingnan Zhang, Lei Li, Lu Chen, Yanhong Zhai, Zheng Cao

**Affiliations:** ^1^ Department of Laboratory Medicine Beijing Obstetrics and Gynecology Hospital Capital Medical University Beijing China

**Keywords:** platelet, preeclampsia, pregnancy, reference interval, thromboelastography

## Abstract

**Background:**

Thromboelastography (TEG) provides global assessment of hemostatic function and has been recommended to monitor potential coagulopathies during pregnancy in which hypercoagulable state is favored. In present study, we established the reference intervals (RIs) of the TEG parameters (R, K, MA, and α‐angle) with Chinese pregnant women of third trimester. In addition, we examined the diagnostic efficacies of the TEG parameters in the patients diagnosed of gestational hypertension (GH), gestational diabetes mellitus (GDM), or preeclampsia (PE).

**Methods:**

With specified including and excluding criteria, non‐pregnant controls, healthy pregnant women, and pregnant women with GH, GDM, or PE had their venous blood drawn at Beijing Obstetrics and Gynecology Hospital, followed by TEG tests performed in the clinical laboratory.

**Results:**

The RIs determined with the healthy pregnant women (in third trimester) for R, K, MA, and α‐angle were 4.0‐7.7, 1.2‐3.2, 51.9‐70.1, and 41.4‐74.4, respectively. When compared with the healthy pregnancy group, the K value was significantly decreased in GH patients but increased in PE patients; MA was significantly lower in the PE group. In the receiver operating characteristic curve (ROC) analyses, K value was able to efficiently distinguish normal pregnancy from the GH patients, with an AUC of 0.86 which is far better than those of R (AUC = 0.57) and MA (AUC = 0.56). For the PE patients, the AUC of MA (0.69) was significantly greater than that of R (0.50).

**Conclusions:**

Thromboelastography may provide more accurate experimental basis for monitoring coagulation functions especially in pregnant women with complications of GH and PE.

## Graphical abstract

Thrombelastography (TEG) provides global assessment of hemostatic function and has been recommended to monitor potential coagulopathies during pregnancy in which hypercoagulable state is favored. Here we established the reference intervals (RIs) of the TEG parameters (R, K, MA and α angle) with Chinese pregnant women of third trimester. In addition, we examined the diagnostic efficacies of the TEG parameters in the patients diagnosed of gestational hypertension (GH), gestational diabetes mellitus (GDM) or preeclampsia (PE). The figure presents the boxplots of the TEG parameters R (A), K (B), MA (C) and α angle (D) in non‐pregnant (NP), healthy pregnancy (HP), gestational hypertension (GH), gestational diabetes mellitus (GDM) and preeclampsia (PE) groups. Student’s *t* test was performed to calculate the p values (^*^ *P* < .05).

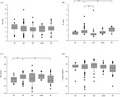

## 1 INTRODUCTION

In the mid‐late stage of pregnancy, the coagulation, anti‐coagulation, and fibrinolytic system were significantly changed with increased circulating levels of clotting factors, decreased natural anticoagulants, and fibrinolytic activity, resulting in a state of hypercoagulability to maintain placental function and ensure the rapid and effective control of bleeding at the time of placental separation.^1^, ^2^, ^3^, ^4^ The pro‐coagulation state is further exaggerated in pregnancy‐related pathological complications, such as preeclampsia (PE), gestational diabetes mellitus (GDM), and gestational hypertension (GH).^5^ Therefore, close monitoring the coagulation function and hemostatic disturbance during pregnancy is of high importance.^4^

Thromboelastography (TEG) provides the real‐time diagram of coagulation ability and records the formation of original clot, platelet activation, and production of fibrin protein.^6^, ^7^ Among the four TEG parameters that are commonly determined in clinical laboratories, reaction time (R time) measures the time interval from the start of the test to the initial detection of the clot. Similar to prothrombin time (PT), R time provides information about factor deficiencies and heparin therapy. The clot strength is measured by two parameters in TEG: K value and α‐angle. Both of the K value and the α‐angle represent clot kinetics and mainly depend on fibrinogen levels; they can help identify states of hyper‐ or hypo‐coagulopathies. Maximum amplitude (MA) is a measurement of maximum clot strength and gives information on both fibrinogen and platelet functions.^8^

As TEG assesses global hemostatic function and is sensitive to coagulopathies,^9^, ^10^, ^11^ it has been widely used in various clinical conditions such as trauma and surgery related dilutional coagulopathy, hemophilia, coronary artery bypass, pregnancy, and postpartum hemorrhage.^12^, ^13^, ^14^

Previous studies have recommended the use of TEG for monitoring hypercoagulation in pregnant women^9^ and formulating the treatment strategy.^10^ However, the non‐pregnant reference intervals (RIs) that are adopted universally in clinical laboratories have been proven to be unsuitable for pregnancy.^11^ There was limited published data and lack of consensus on the RIs for standard TEG application in late pregnancy.^12^ Hence, in present study, we established the RIs of the TEG parameters (R, K, MA, and α‐angle) with Chinese pregnant women of third trimester. In addition, we examined the relative changes and the diagnostic efficacies of the TEG parameters in the patients diagnosed of GH, GDM, or PE.

## 2 MATERIALS AND METHODS

### 2.1 Subjects

Healthy pregnant women in third trimester attending routine antenatal check‐ups in the Beijing Obstetrics and Gynecology Hospital were initially included. The exclusion criteria for healthy pregnant subjects were as follows: (1) below 18 year or above 45 year of age; (2) a medical history of coagulopathy and/or thromboembolic disease; and (3) anti‐coagulation treatment and/or treatment with anti‐platelet drugs during pregnancy.

The GDM patients were diagnosed by the universal screening at 24‐28 weeks of gestation using 75 g of glucose in a 2‐hour oral glucose tolerance test (OGTT) according to the International Association of Diabetes and Pregnancy study Groups (IADPSG) 2010 criteria.^13^ Specifically, GDM was defined by meeting at least one of the three following criteria: FPG ≥5.1 mmol/L, 1‐hour postprandial blood glucose ≥10.0 mmol/L, and 2‐hour postprandial blood glucose ≥8.5 mmol/L. The preeclampsia diagnosis was determined with the diagnostic criteria proposed by the 2019 ACOG Practice Bulletin,^14^ in which preeclampsia was defined as gestational hypertension (systolic/diastolic blood pressure ≥140/90 mm Hg) in previously normotensive women accompanied by proteinuria (urine protein ≥300 mg/24 hours) or end organ damage after 20 weeks of gestation.

Meanwhile, healthy non‐pregnant women of 18‐45 years old were recruited as controls in present study. The exclusion criteria for the control group were as follows: (1) a medical history of coagulopathy and/or thromboembolic disease; (2) anti‐coagulation treatment and/or treatment with anti‐platelet drugs in the last 30 days; (3) having taken hormonal contraceptives in the last 6 months; and (4) having undergone delivery or abortion in the last 6 months.

This study was approved by Ethnics Committee of the hospital (approval number 2017‐KY‐083‐01), and all participants included in the study signed consent forms.

### 2.2 Methods

The recruited healthy pregnant women and the pregnant women with GH, GDM, or PE had their venous blood drawn in their third trimester (29‐40 weeks of gestation). For routine coagulation testing panel, the blood sample of each subject was collected into a tube containing 0.11 mmol/L (3.2%) sodium citrate (one part of anticoagulant plus nine parts of blood), followed by centrifugation at 1800 *g* for 10 minutes. The coagulation tests including prothrombin time (PT) (catalog: OWHM13), activated partial thromboplastin time (APTT) (catalog: B4219‐2), thrombin time (TT) (catalog: OUHP49), D‐dimer (DD) (catalog: OPBP07), and fibrinogen (FIB) (catalog: B4233‐27) were performed on the Sysmex CS 5100 automatic coagulation analyzer Sysmex Corporation, Kobe, Japan). For platelet count (PLT), the routine complete blood count (CBC) analysis was performed on the Sysmex XN‐2000/3000 automatic blood cell analyzer (Sysmex Corporation, Kobe, Japan) following the standard operation procedure recommended by the manufacturer.

The TEG experiments were performed on the TEG 5000^®^ Thrombelastograph analyzer (Hemostasis System, USA). Briefly, 1 mL of citrated whole blood sample was added into the kaolin activator bottle. Then, 340 μL of the inverted sample is mixed with 20 μL of 0.2 mmol/L calcium chloride in a testing cup. The values of the four TEG parameters were directly recorded from the instrument.

### 2.3 Statistical analysis

SPSS Statistic 21 (SPSS Inc, Chicago, IL, USA, RRID:SCR_002865) was used in boxplots preparation and receiver operating characteristic curve (ROC) analyses. The RIs of TEG parameters with normal pregnant women of third trimester were presented as 2.5‐97.5th percentiles according to the Clinical and Laboratory Standards Institute (CLSI) guideline EP28‐A3C.^15^ Pearson's test was used to examine the correlation between thromboelastographic parameters and PLT or conventional coagulation tests. ROC was applied to evaluate the diagnostic efficacy of each TEG parameter by calculating the area under the curve (AUC). A *P* value of less than .05 was considered to be statistically significant.

## 3 RESULT

### 3.1 Patient enrollment

With the recruiting and excluding criteria described in the Method section, from July to December of 2019, totally 125 healthy women in their third trimester of physiological pregnancy were enrolled for the establishment of RIs of TEG parameters. Meanwhile, 20 healthy non‐pregnant women, 70 patients diagnosed with PE, 40 with GDM, and 50 with GH were recruited as controls or comparing groups.

### 3.2 Reference intervals of TEG parameters in late pregnancy

The RIs determined with the healthy third trimester pregnant women for R, K, MA, and α‐angle were 4.0‐7.7 (minutes), 1.2‐3.2 (minutes), 51.9‐70.1 (mm), and 41.4‐74.4 (degree), respectively (Table 1). The 90% confidence intervals of the TEG RIs were also listed in Table 1.

**Table 1:** The reference intervals of thromboelastogram parameters in late pregnancy

	R	K	MA	α‐angle
n = 125, mean age = 31.9				
2.5% percentile (90% CI)	4.0 (3.8, 4.3)	1.2 (1.2, 1.3)	51.9 (50.2, 53.2)	41.4 (36.3, 56.3)
97.5% percentile (90% CI)	7.7 (7.0, 9.5)	3.2 (2.6, 4.5)	70.1 (69.5, 70.7)	74.4 (72.2, 74.8)

R: r time (min); K: k‐time (min); MA: maximum amplitude (mm); α‐angle (degree); CI, confidence interval.

### 3.3 Comparison of TEG parameters in different groups

**Figure 1:**
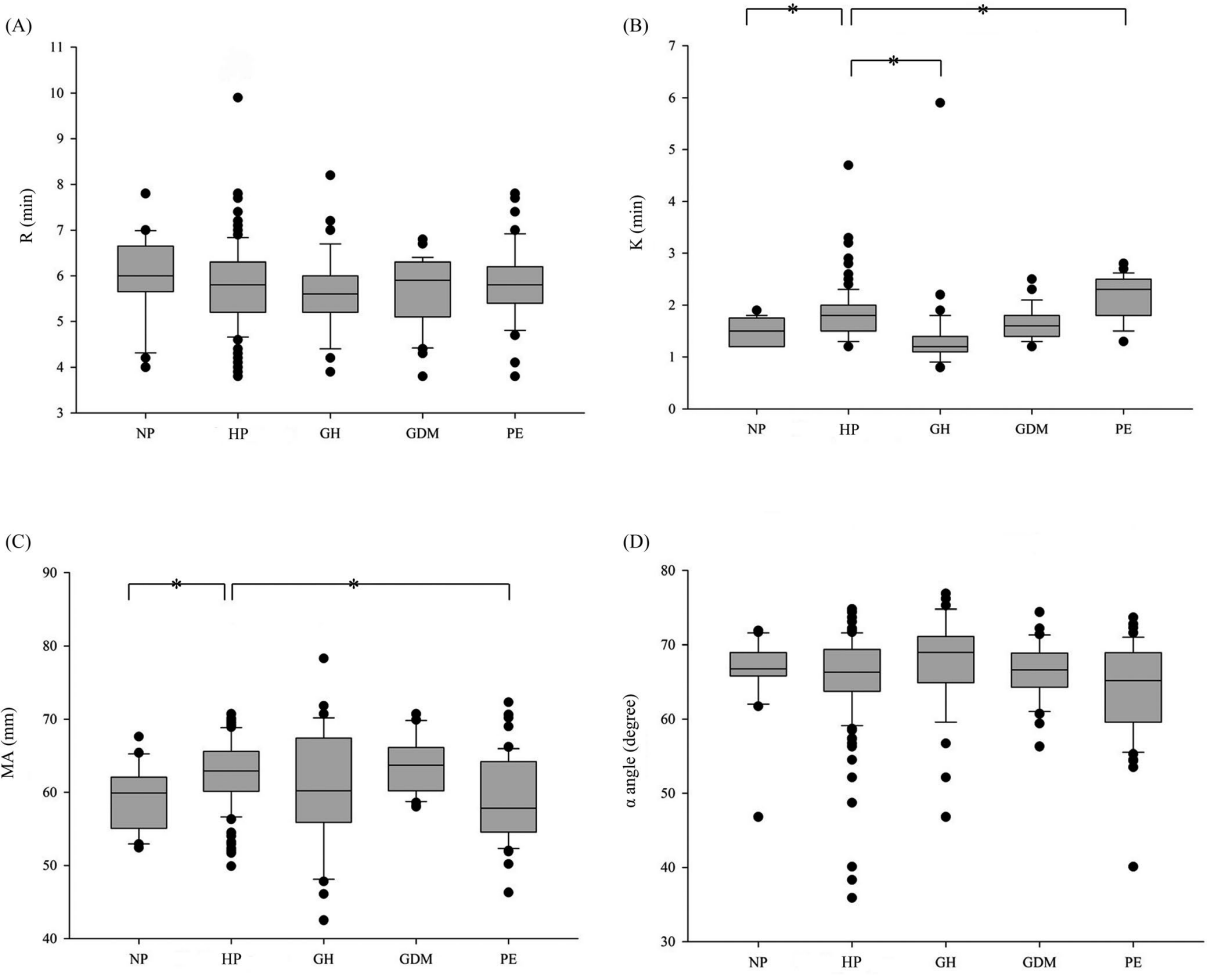
Boxplots of the TEG parameters R (1A), K (1B), MA (1C), and α‐angle (1D) in non‐pregnant (NP), healthy pregnancy (HP), gestational hypertension (GH), gestational diabetes mellitus (GDM), and preeclampsia (PE) groups. The bottom and top of the box represent the 25th and 75th percentile. The band inside the box shows the median. Student's *t* test was performed to calculate the p values (*P < .05)

As shown in Figure 1B,C and Table S1, the means of K and MA were significantly higher in the healthy pregnant women than in the non‐pregnant controls, suggesting the necessity of establishing pregnancy specific RIs for TEG parameters. However, for R time and α‐angle, when compared with the healthy pregnancy group, there was no statistical difference in non‐pregnant control, GH, GDM, or PE groups (Figure 1A,D, and Table S1). Impressively, the K value was significantly decreased in the GH patients (*P* = .003) but increased in the PE patients (<0.001); MA was significantly lower in the PE group than in normal pregnancy (Figure 1 and Table S1).

### 3.4 ROC analyses

**Figure 2:**
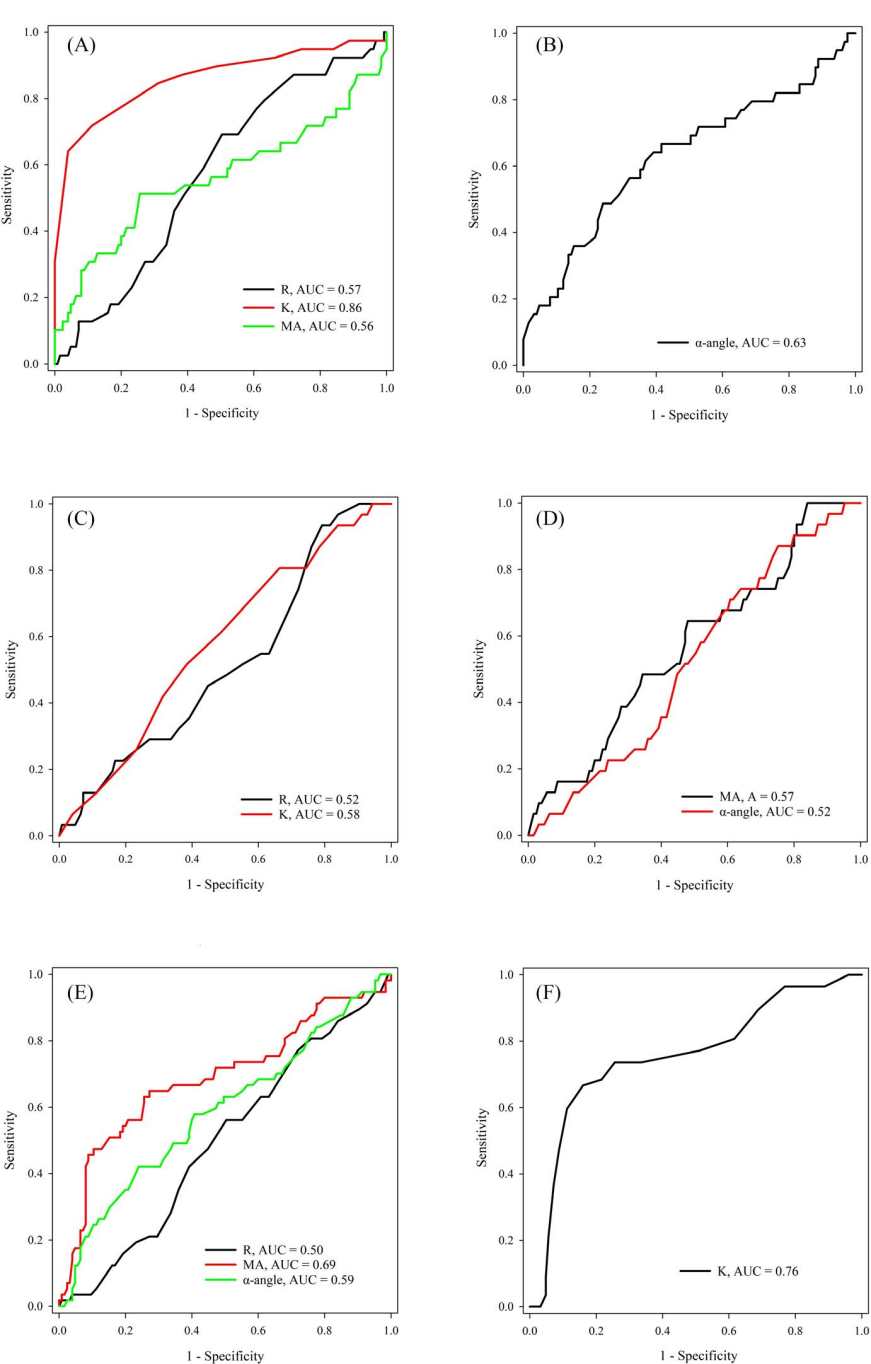
ROC analyses of TEG parameters (R, K, MA, and α‐angle) in the diagnosis of gestational hypertension (GH) (A, B), gestational diabetes mellitus (GDM) (C, D), and preeclampsia (PE) (E, F). In Figure 2A, C andE, the TEG parameters were decreased in GH, GDM or PE patients; In Figure 2B, D, andF, the TEG parameters were increased in GH, GDM, or PE patients

To evaluate the diagnostic efficacies of TEG parameters in various pregnancy complications including GH, GDM, and PE, the ROC analyses were performed and the AUCs of each TEG variables were compared. As indicated in Figure 2, K, R, and MA were decreased in GH patients (Figure 2A); K value was able to efficiently distinguish normal pregnancy from the GH group, with an AUC of 0.86 which is far better than those of R (AUC = 0.57) (*P* < .05) and MA (AUC = 0.56) (*P* < .05). On the contrary, α‐angle was increased in GH patients with an AUC of 0.63 (Figure 2B). For the PE patients (Figure 2E), MA, R, and α‐angle were decreased; the AUC of MA (0.69) was significantly greater than that of R (0.50) (*P* < .05). The K value was increased in the PE patients with an AUC of 0.76 (Figure 2F). However, in the ROC analyses with the GDM patients, there was no statistical difference in the AUCs between R and K (decreased in GDM, Figure 2C) or between MA and α‐angle (increased in GDM, Figure 2D).

### 3.5 Correlation between TEG parameters and conventional coagulation indices or platelet count

The correlation between the TEG parameters and conventional coagulation tests (including PT, APTT, TT, FIB, and DD) was assessed with the Pearson's correlation method in the healthy pregnancy group. As shown in Table S2, there was no significant correlation between any of the TEG parameters and routine coagulation indices. In the correlation studies between MA and platelet count performed with the four pregnant groups (healthy, GH, GDM, and PE), the MA values were essentially plateaued across the entire platelet count range (100‐400*10^9^/L) observed in present study (Figure S1A‐D).

## 4 DISCUSSION

Thromboelastography had emerged in many settings as reliable means to urgently assess coagulation status in pregnancy. As a vital factor influencing the TEG results, the RIs in pregnant population have not been well characterized. In this study, we established the RIs for the TEG parameters R, K, MA, and α‐angle with healthy pregnant women of third trimester. When compared with the non‐pregnant control group, the R was decreased (although not statistically significant) and the MA was significantly increased (Table S1), which was consistent with the hypercoagulable state in pregnancy. Similar findings were also observed in other studies with TEG^15^ or ROTEM^®^ (TEG equivalent instrument)^16^ in late pregnancy. However, unlike the previous report in which K was found decreased in Caucasians,^15^ the K value was slightly increased in our pregnant group (Figure 1B). This apparent discrepancy may be attributed to the relatively small group size of non‐pregnant subjects in our study and/or the significance of Asian ethnicity background.

Variations of TEG testing results between healthy and abnormal pregnant women were observed in our study (Figure 1 and Table S1). For instance, in the GH group, the R and K values were decreased while the MA and α‐angle values were increased, indicating hyperactive states of clotting factors and fibrinogen levels.^17^, ^18^ Previous study showed that many of the pathophysiologic changes of hypertension diseases during pregnancy could be initiated by perturbed endothelial cell function, due to increased production of fibronectin and coagulation cascade proteins.^19^ By contrast, none of the TEG parameters in the GDM group were significantly different from those in healthy pregnant women. Interestingly, the article by Shupletsova et al reported that the GDM patients experiencing cerebral ischemia (CI) in newborn infants were more likely to present decreased R and K and increased MA and α‐angle than the GDM patients who ended up with no obvious adverse outcomes, implying that glycemia during pregnancy may be involved in programming the development of coagulation disorders and could have effects on TEG results. Unlike GH and GDM, more studies were focused on the direct comparison of TEG parameters between normal and PE pregnancies.^16^, ^20^, ^21^ Interestingly, completely opposite changes of TEG parameters were observed in different studies. In the work by Wang et al,^16^, ^20^ the mean values for R and K among the women with PE were higher than those of the control group; the mean values for MA and α‐angle were lower in the PE group than in the control group. These findings were similar to ours (Figure 1 and Table S1), implying coagulation damage that may be related with increased tissue factor and consumption of clotting factors in PE patients.^16^ However, according to the study by He et al,^21^ the R and K values were found decreased while the MA and α‐angle were increased in PE group, which was consistent with a hypercoagulable state although sampling time was not clearly indicated for PE patients. Therefore, future research with strict sampling window and larger patient cohort is warranted to reveal the dynamic changes of TEG parameters in pregnancies with or without complications.

Although it has been reported that TEG parameters were correlated with some conventional coagulation indices in a healthy pregnant group (n = 566) in which 68.1% of the subjects were >37 gestational weeks,^22^ no similar observation was made with our data. Interestingly, it has been previously reported that MA was positively correlated with PLT in normal pregnancy^22^ especially when PLT < 100*10^9^/L,^22^ suggesting that TEG is sensitive to PLT quantitative or qualitative abnormalities. When PLT > 100*10^9^/L, however, the MA value was plateaued and became insensitive to the change of PLT^22^ which was essentially the same as our results (Figure S1A‐D).

## 5 CONCLUSION

In summary, the RIs of the TEG parameters in healthy pregnant women were established in this study. Compared with routine coagulation tests, TEG may provide more accurate experimental basis for monitoring coagulation functions especially in pregnant women with complications of GH and PE.

## CONFLICTS OF INTEREST

The authors declare no conflict of interest. The sponsor had no role in the design, execution, interpretation, or writing of the study.

## Supporting information

Figure S1

Table S1‐S2
