# Supplementary figures and images for: Thromboelastography (TEG) in normal pregnancy and its diagnostic efficacy in patients with gestational hypertension, gestational diabetes mellitus, or preeclampsia
Source: J Clin Lab Anal. 2020 Oct 17;35(2):e23623. doi: 10.1002/jcla.23623 (PMC7891543; doi:10.1002/jcla.23623)

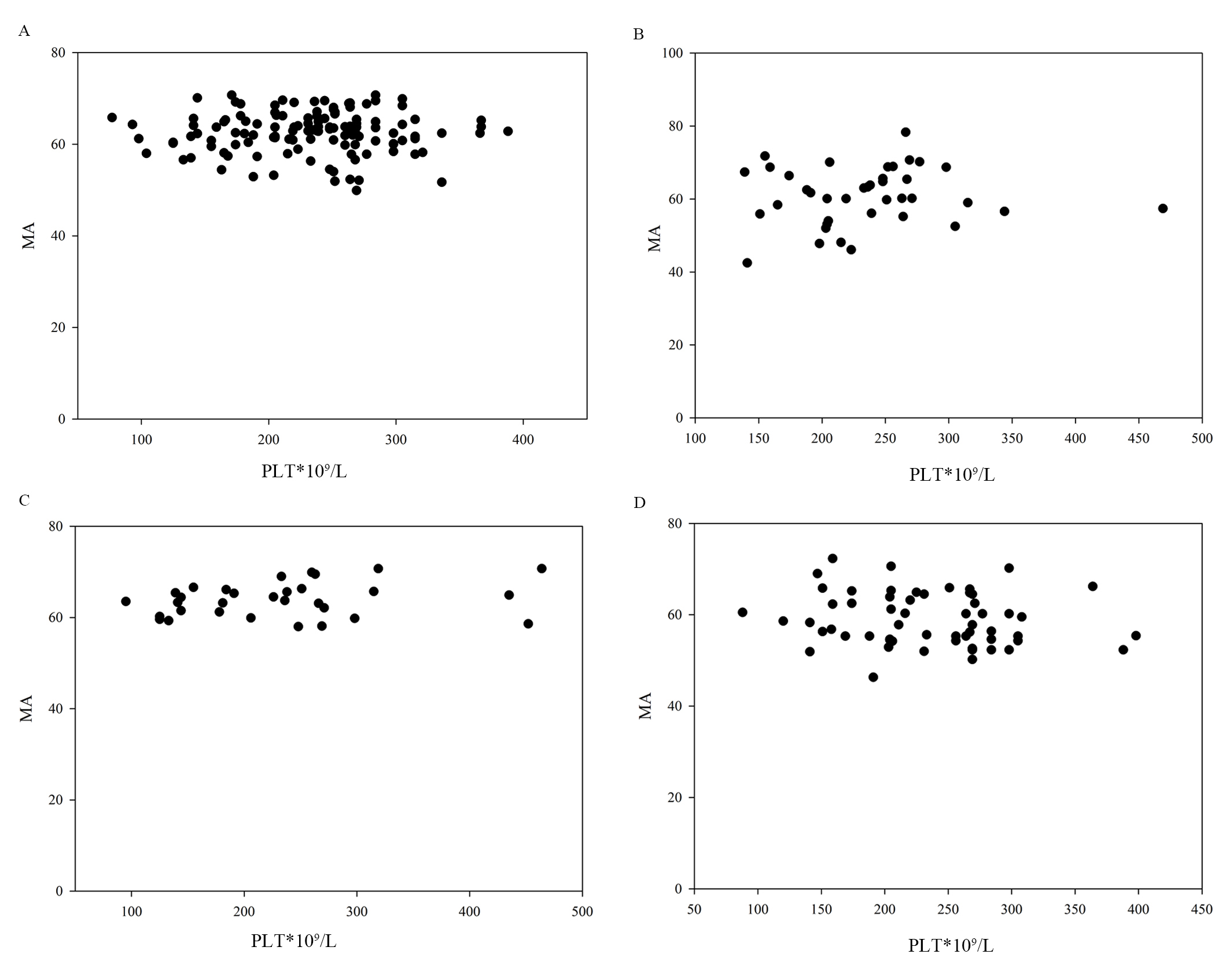

Supplement: Supplementary file 1 — Figure S1 [file JCLA-35-e23623-s001.jpg]
