# Supplementary material for: Thromboelastography (TEG) in normal pregnancy and its diagnostic efficacy in patients with gestational hypertension, gestational diabetes mellitus, or preeclampsia
Source: J Clin Lab Anal. 2020 Oct 17;35(2):e23623. doi: 10.1002/jcla.23623 (PMC7891543; doi:10.1002/jcla.23623)
Supplement: Supplementary file 2 — Table S1‐S2 [file JCLA-35-e23623-s002.docx]

**Supplementary Tables**

**Supplementary Table 1** Comparison of TEG parameters between different patient groups

|  |  | R | K | MA | α angle |
| --- | --- | --- | --- | --- | --- |
| Mean | non-pregnant (NP) | 6.0 | 1.5 | 59.1 | 66.4 |
|  | healthy pregnancy (HP) | 5.8 | 1.8 | 62.6 | 65.4 |
|  | GH | 5.6 | 1.4 | 60.9 | 67.6 |
|  | GDM | 5.7 | 1.7 | 63.9 | 66.4 |
|  | PE | 5.8 | 2.2 | 58.9 | 63.8 |
| p values of different comparison groups | NP vs HP | 0.341 | <0.001 | 0.003 | 0.455 |
|  | GH vs HP | 0.260 | 0.003 | 0.213 | 0.068 |
|  | GDM vs HP | 0.453 | 0.061 | 0.108 | 0.275 |
|  | PE vs HP | 0.843 | <0.001 | <0.001 | 0.121 |
| GH: gestational hypertension; GDM: gestational diabetes mellitus; PE: preeclampsia. | | | | |  |

**Supplementary Table 2** Correlation between TEG and routine coagulation test in healthy pregnancy of third trimester

|  |  |  |  |  |  |  |  |  |  |  |  |  |  |  |
| --- | --- | --- | --- | --- | --- | --- | --- | --- | --- | --- | --- | --- | --- | --- |
| Variables |  | R | |  | K | |  | MA | |  | α angle | |  |  |
|  |  | *r* | *p* |  | *r* | *p* |  | *r* | *p* |  | *r* | *p* |  |  |
| PT |  | 0.036 | 0.693 |  | -0.051 | 0.571 |  | 0.060 | 0.509 |  | 0.022 | 0.804 |  |  |
| APTT |  | 0.014 | 0.875 |  | 0.012 | 0.897 |  | 0.090 | 0.316 |  | -0.018 | 0.844 |  |  |
| TT |  | 0.003 | 0.974 |  | 0.009 | 0.920 |  | 0.091 | 0.312 |  | 0.063 | 0.485 |  |  |
| FIB |  | 0.119 | 0.185 |  | 0.085 | 0.347 |  | 0.071 | 0.432 |  | -0.156 | 0.082 |  |  |
| DD |  | -0.052 | 0.567 |  | -0.028 | 0.753 |  | -0.013 | 0.883 |  | -0.047 | 0.601 |  |  |
| PLT |  | 0.148 | 0.099 |  | -0.100 | 0.266 |  | -0.005 | 0.959 |  | 0.014 | 0.877 |  |  |
| PT, prothrombin time; APTT, activated partial thromboplastin time; TT, thrombin time; FIB, fibrinogen; DD, D-dimer; PLT, platelet. | | | | | | | | | | | | | | |
| The correlation coefficient (r) and the p values were calculated with the Pearson's correlation method. | | | | | | | | | | | |  |  |  |
|  |  |  |  |  |  |  |  |  |  |  |  |  |  |  |
